# Innovations in the climate assessment development process

**DOI:** 10.1007/s10584-025-04023-1

**Published:** 2025-10-01

**Authors:** Allison R. Crimmins, Christopher W. Avery, David R. Reidmiller, Aaron M. Grade

**Affiliations:** 1 National Oceanic and Atmospheric Administration, 1315 East West Hwy, SSMC III, Silver Spring, MD 20910 USA; 2 Independent Researcher, Cambridge, MA US; 3 Gulf of Maine Research Institute, 350 Commercial St, Portland, ME 04101 USA; 4 ICF, 1902 Reston Metro Plaza, Reston, VA 20190 USA

**Keywords:** Climate change, Assessment, Science communication, Climate policy, Climate services

## Abstract

Climate assessments have long been key scientific inputs that inform the development of productive and impactful climate policy in the United States and around the world. This introduction sets the stage for the suite of papers in the Topical Collection “Advancements in U.S. Climate Assessments.” Inspired and informed by the release of the Fifth National Climate Assessment, the papers within this issue document lessons learned over the past 30+ years and leverage the perspectives of previous assessment authors and staff to aid those interested in developing their own climate assessments. This paper reviews the evolution of climate assessments and the factors that make for useful, usable, and used scientific products to support societal choices. Evolving user needs over the last 30+ years also reflect a shift in demand towards more localized or more context-specific climate data that integrates social science information, tools, and frameworks. To meet these needs, we highlight three areas of potential opportunity and challenge for future assessments: continued and strengthened conversations between assessment developers across geographic scale to share innovations and lessons learned in the development process; working with knowledge holders in under-represented areas of expertise to alter assessment governance and guidelines to better incorporate diverse perspectives; and seizing opportunities for using innovative communication and engagement mediums.

## Introduction

Over the past 30+ years, climate science assessments have become critical inputs to the policies, decisions, negotiations, investments, communications, and actions that rely on best-available science. The United States national assessment process began in 1990, initiating production of the National Climate Assessment (NCA) with the formation of the U.S. Global Change Research Program (USGCRP) (GCRA 1990). Building upon the improvements and innovations of each successive assessment cycle, the NCA has since grown into one of the U.S. government’s most authoritative and useful scientific reports (for a brief history of the development of NCA, see the supplemental materials in Avery et al. 2025).

In 2016, *Climatic Change* published a Topical Collection titled “The US National Climate Assessment: Innovations in Science and Engagement” (Jacobs et al. 2016a). That Topical Collection discussed key lessons learned in the assessment development process, particularly from the Third National Climate Assessment (NCA3), which was released in 2014 (USGCRP 2014). In the nine years since the 2016 Topical Collection was published, climate assessments have continued to undergo an evolution in terms of the scientific content included in the reports, the assessment development process, and public engagement efforts. This new Topical Collection, “Advancements in U.S. Climate Assessments,” seeks to capture those updates and document further evolution of the climate assessment development process, including how changes in scientific knowledge have reshaped the structure and content of climate assessment products.

“Advancements in U.S. Climate Assessments” describes lessons learned from 30+ years of climate assessments, with an emphasis on the U.S. NCAs. The papers in this Topical Collection also highlight advancements in the assessment development process at state, regional, and international scales, and reflect on best practices and future directions for other scientific assessments. The Topical Collection showcases innovative visions from leaders in the assessment community, identifies constraints and challenging tradeoffs inherent throughout the development process, and highlights opportunities to transform future assessments to better meet user needs. While the papers in this collection may highlight changes in general scope or growth into new content areas, they do not summarize or evaluate the content or findings of previous climate assessments. Instead, these papers focus on advancements in the assessment development purpose, process, and products. The goal is to highlight innovations and share best practices such that governments or organizations developing— or considering developing—climate assessments can deliver the most societally valuable products possible.

### Timely reflection

The release of the Fifth National Climate Assessment (NCA5; http://nca2023.globalchange.gov) (USGCRP 2023c) was announced by President Biden in November 2023 (EOP 2023). This assessment will be the report of record until the Sixth National Climate Assessment is published, expected in 2028 (https://globalchange.gov/nca6), just shy of 40 years since the Global Change Research Act (GCRA) was first proposed to the Senate in January 1989 (GCRA 1990). After five NCAs—as well as an increasing number of climate assessment efforts in other countries and at local, state, regional, and global scales—the time is ripe to reflect on how assessments have evolved over time and how assessment developers can design future processes, structures, and engagement strategies to best serve the unique needs of users while maintaining the highest levels of scientific integrity.

Some climate assessments, such as the Intergovernmental Panel on Climate Change (IPCC) at the global level, the NCA at the national level, and the California State Climate Change Assessment at the state level, have already undergone several development cycles and built institutional systems and processes over time to address challenges (Avery et al. 2025). These institutions have benefited from and built upon lessons learned in early assessment cycles to improve responsiveness to user needs; manage resource constraints (e.g., staffing, time, budget); ensure robust, transparent, and authoritative products; navigate new or evolving governance structures and statutory requirements; and effectively communicate the state of the science to decision-makers. The experience gained over these iterative development cycles can inform other assessment developers, especially those being initiated by regional, state, Tribal, and local governments for use in adaptation planning, risk management, and decision-making. At the same time, climate assessments that are in their first or second cycle of development, such as Puerto Rico, the U.S. Virgin Islands, and the *Status of Tribes and Climate Change* (STACCWG 2025), are demonstrating novel report structures and formats, as well as innovative ways to include and integrate social sciences (Maxwell et al. 2025). This creativity, which may in fact stem from the lack of precedent or institutional expectations of what a climate assessment looks like, can inspire innovations for other assessment developers, especially those shifting away from reporting only geophysical findings and towards comprehensive delivery of climate services.

USGCRP and other climate assessment developers now find themselves at a critical inflection point: The seventh round of the IPCC Assessment Reports (IPCC 2023b) and the sixth round of the National Climate Assessment (NCA6) (USGCRP 2024a) are in early stages of development. At the same time, more and more U.S. states are ramping up the next cycle of their next assessments (e.g., California 2018; Wuebbles et al. 2021) while metropolitan departments of transportation and planning organizations plan their first climate vulnerability assessments (Whitehead et al. 2023). The second volume of the Status of Tribes & Climate Change Report was recently released (STACCWG 2025). Outside the U.S., many countries are taking on their first climate risk and vulnerability assessment as part of the United Nations guidance on formulation and implementation of National Adaptation Plans (Dave et al. 2025); UNFCCC 2024; for example, see DCCEEW 2024). With the growth in interest and investment in climate services (see, for example, NSTC 2023), there are new opportunities arising for programs like USGCRP to connect decision-makers at the federal, state, local, and Tribal levels with subject matter expertise (NASEM 2021). With the community of assessment developers expanding so rapidly, the contributors to this Topical Collection strive to share insights and open a wider dialogue on how to continue to improve the assessment development process.

### Advancements in assessment development covered in this Topical Collection

This Topical Collection is geared towards a similar audience as the 2016 Topical Collection but a different audience than that of the NCA or other assessments themselves. The audience for these Topical Collection papers are namely “those sponsoring, designing, and assisting in assessments at regional, national, and international levels” (Jacobs et al. 2016b), with a goal of inspiring discussion on how assessments can improve science communication and be made more useful to a broad range of decision-makers. This Topical Collection documents best practices, lessons learned, and successful operational building blocks that create the foundation for valuable assessment reports—the who, what, where, when, how, and why of conducting assessments—while also illustrating how scientific and societal advances over the past several decades have necessitated changes in both the products and process. Such documentation of lessons learned and challenges faced is valuable as future assessments continue to grow in number and evolve in scope.

This Topical Collection is broken into four primary sections that encompass a wide range of topics related to advancements in climate assessments, written by authors with expertise on developing international, national, state, and local assessments. The articles communicate the importance of assessments and highlight advances in key elements of development. The first section is on inclusion of diverse voices in climate assessments. This includes “Brief History of Indigenous Involvement in the National Climate Assessment,” (Whyte et al. 2025) which reviews established best practices for respectful inclusion of Indigenous Knowledges, authors, and perspectives in climate assessments; “Centering Environmental Justice in United States (U.S.) National Climate Assessments (NCAs): A Historical and Contemporary Analysis” (Méndez et al. 2025) which discusses how the conversation around climate change has evolved from a focus on the primary physical drivers of climate change to discussion of impacts and response from an environmental justice lens; and a letter on “Broadening Diversity, Equity, Accessibility, and Inclusion in the Process and Development of Climate Assessments,” (Chu et al. 2025) which outlines the importance of inclusion of a diverse set of contributors and audiences to climate assessments.

The second section covers scientific advances in the scope of climate assessments, including “Projections of Future Climate for U.S. National Assessments: Past, Present, Future,” (Basile et al. 2025) which summarizes the latest science on downscaling and climate projections to inform decisions around inclusion of modeling outputs in future assessments. “The Social Sciences in Climate Assessments in the United States” (Maxwell et al. 2025) covers the opportunities and challenges inherent in the expansion of social science disciplines in climate assessments. A paper titled “Incorporating Research Gap Identification Processes into Climate Change Assessments: A California Case Study for Localized Research and Knowledge” (Matouka and Basile 2025) demonstrates recent approaches for identifying gaps in research via the assessment process, highlighting the California State Climate Change Assessment as an iterative process of identifying research needs, funding studies to address those unknowns, and reporting results. “Analysis of Nature-Related Themes and Terminology in U.S. Climate Assessments” (Conrad-Rooney et al. 2025) discusses the evolution of coverage and definitions of nature-related topics and terminology across NCAs.

The third section of the Topical Collection is focused on advancements in the assessment development process. “Navigating complex waters: Designing a process for the development of the National Climate Assessment” (Avery et al. 2025) is a comprehensive look at how the NCA5 development process met complex statutory and user-driven requirements and provides insights into the tradeoffs inherent in designing an assessment. “Public Engagement in Climate Assessment: Lessons and Opportunities” (Lustig et al. 2025a) highlights the ways that various assessments have approached public engagement to maximize user utility and connect to diverse audiences. This section also includes a letter titled “Bringing Art and Science Together to Address Climate Change.” (Lustig et al. 2025b). 

The fourth section of the Topical Collection looks at advances in assessments across different geographies: in addition to the state level proceses explored in Matouka and Basile (2025), these papers span from a sub-national regional chapter of the NCA to assessments developed by other countries and regions outside the United States. “Reflections on Preparing Regional Chapters for the NCA5” (Frazier et al. 2025) looks at the development of multi-state regional chapters in NCA5, highlighting the importance of these chapters for informing local-scale decision-making and providing recommendations to inform future assessments. “Reflections on International Climate Assessments” (Dave et al. 2025) compares examples of national and multi-nation regional climate assessments around the world .

In the remainder of this scene-setting contribution, we review the stated goals in the conventions and laws establishing climate assessments to provide context for the historical purpose behind the development of these reports and to highlight how that purpose has evolved as climate science has advanced. We then describe the guiding principles of all USGCRP assessments and emphasize the specific goals for NCA5, which are reflected in many of the other papers within this Topical Collection. Finally, we highlight three areas of potential opportunity and challenge for future assessments as they seek to continuously meet evolving user needs.

## The value of scientific assessments

While expert communities beyond the field of climate change develop ad hoc or periodic scientific assessments (for example, IPBES 2019; USGCRP 2022; WMO 2022), reports of the scale and magnitude of climate assessments conducted with institutional continuity are rare in other areas of study, especially beyond the field of global environmental change (Oppenheimer et al. 2019). Global environmental assessments have been defined as “large-scale social processes where large groups of experts convene to interpret, deliberate and synthesize existing scientific knowledge on complex environmental issues with a view to inform public policy” (Kowarsch and Jabbour 2017). Definitions of global environmental assessments commonly emphasize the “large” nature of these processes, noting the increasingly elaborate and formal development structures. Why, then, are scientific assessments common in environmental science and especially climate science, particularly when their development requires such a significant level of time, effort, and resources? We posit that the answer is due to the value of global environmental change assessments, which itself is due to several connected factors.

The first factor is the nature of the problems or threats being assessed. Oppenheimer et al. (2019) notes society’s reliance on “scientists as sentinels”, particularly for issues like acid rain and climate change, because “laypeople” (those without specialized knowledge and expertise) are not in a position to understand or recognize such threats. Climate change and other global changes may be realized over large temporal and geographic spans and combine with other factors to result in a range of impacts, making it difficult for non-experts to identify it as a threat. Climate change has also often been termed a “wicked problem” because of the complexity, interdependencies, and conflicts associated with the response required to address such a global environmental threat (e.g., Lazarus 2009).

This leads to the second factor: the numerous and complex solutions required to address the threat of global environmental change take place at the science-policy interface (Jabbour and Hunsberger 2014; Kowarsch 2016). The threats outlined in scientific assessments are often those that require international cooperation and multiple sustained interventions rather than a single “silver bullet” remedy. Many of the earliest scientific assessment endeavors stem from intergovernmental organizations like the Organisation for Economic Co-operation and Development (see for example the Assessment of Long-Range Transport of Air Pollutants: OECD 1977) and the UN Framework Convention on Climate Change (UNFCCC). Such intergovernmental bodies enact policy and finance responses to complex global change issues based on assessment findings: OECD’s assessment on long-range transport of air pollutants, for instance, informed critical acid rain policies while the IPCC assessment reports inform international climate negotiations and agreements. Scientific assessments also inform national and sub-national policies, research agendas, and social movements (Oppenheimer et al. 2019).

Thus, scientific assessments mobilize knowledge production around high-stakes societal risks and deliver it to those acting to address environmental threats. This leads to the third factor that makes global environmental assessments, and especially climate assessments, so valuable and prevalent: they serve as the bridge between science and action. For climate change in particular, this has involved reducing real and perceived uncertainty in the science enough to justify action. Around the same time frame that the IPCC was being established, U.S. President George H.W. Bush signed the GCRA into law. The text of the GCRA notes that the formation of the USGCRP is in part driven by the need for better science on global change to inform effective policy: “Development of effective policies to abate, mitigate, and cope with global change will rely on greatly improved scientific understanding of global environmental processes and on our ability to distinguish human-induced from natural global change” (GCRA 1990).

In addition to improving detection and attribution, the GCRA also mandates that USGCRP develops assessments that integrate, evaluate, and interpret its findings “and [discuss] the scientific uncertainties associated with such findings.” In response to this mandate, NCA has adopted practices similar to the IPCC around the use of calibrated language to describe and quantify levels of uncertainty (Mastrandrea et al. 2011; Crimmins 2020). Like the IPCC reports, the NCAs have made increasingly confident statements over the years around the connection between anthropogenic emissions and climate impacts. Scientific consensus on this topic is so strong it necessitated development of new calibrated language terminology to express such high levels of certainty. NCA5, for instance, states: “It is *unequivocal* that human activities have increased atmospheric levels of carbon dioxide and other greenhouse gasses. It is also *unequivocal* that global average temperature has risen in response” (Marvel et al. 2023). This example highlights the value of large-scale collaborative efforts convened to synthesize the state of the science—enabling any reader to assess advances in our understanding over time. Even as researchers continue to refine understanding of Earth’s climate system, detail specific climate risks, and update scenarios encompassing different response options, the scientific and policy communities now have adequate information to assess anthropogenic attribution and to inform mitigation and adaptation decisions. The science is well established and sufficiently actionable (USGCRP 2023c).

In fact, scientific understanding of global environmental processes has unquestionably improved significantly since 1990 (e.g., Leung et al. 2023; Forster et al. 2021). Furthermore, the state of the science has evolved from seeking to better quantify specific radiative forcers (i.e., anthropogenic emissions of greenhouse gases and alterations to land use; e.g., Arias et al. 2021; Fahey et al. 2017) to understanding the magnitude, timing, and disproportionate impacts of climate change (e.g., Sherwood et al. 2020; Marvel et al. 2023; IPCC 2021; Eyring et al. 2019), as well as society’s role in both driving and responding to climate change (e.g., Dietz et al. 2020; USGCRP 2022; Pfleiderer et al. 2025). Contemporary assessments are less focused on fundamental processes that are now well-characterized and increasingly focused on reducing uncertainty in low-probability/high-impact effects (e.g., tipping points, complex and compound events; Kopp et al. 2017; Singh et al. 2023), the role of transformational social responses (e.g., changes in human behavior and governance; Maxwell et al. 2025; 2022; Marino et al. 2023), and equity (e.g., cascading and cumulative impacts, just transitions; Méndez et al. 2025; IPCC 2023a; USGCRP 2023c). In addition, the framing of climate assessments has shifted over the years from describing uncertainties in Earth system responses to identifying climate change as a risk management problem by describing the full range of potential impacts and providing accessible, actionable information that decision-makers need to make choices around those risks (NASEM 2016; Weaver et al. 2017; Sutton 2018).

There is a fourth and sometimes overlooked factor involved in the value of global environmental assessments. Instead of thinking about scientific assessments in terms of the reports produced, they are better understood as social processes that collectively inform and connect the scientific community, policymakers, and society (Kowarsch and Jabbour 2017). One potentially underappreciated outcome of assessment work is that it intentionally brings together an increasingly diverse group of multidisciplinary experts that otherwise may not find the impetus or opportunity to collaborate (Chu et al. 2025; Maxwell et al. 2025; Whyte et al. 2025). Indeed, cross-disciplinary collaboration is increasingly contributing to the successful design of lasting solutions to complex climate challenges.

For the NCA, USGCRP creates a working environment in which people from a diversity of disciplines—natural scientists, social scientists, practitioners, Indigenous Knowledge holders, etc.—and a range of sectors—government, academic, private, nonprofit, etc.—work together on a multiyear project. For example, USGCRP provides peer-to-peer training on topics that may be unfamiliar to authors with different areas of expertise. Over multiple rounds of review, NCA author teams work together to determine what content to cover within limited chapter space and must concur on their findings. USGCRP then fosters cross-chapter coordination, through mechanisms like facilitated one-on-one discussion between chapter leads, all-author meetings, and targeted topical reviews (e.g., Conrad-Rooney et al. 2025). This type of long-term, consensus driven collaboration fosters respect across scientific disciplines, provides opportunities to learn from colleagues with different perspectives, and develops new lines of communication across sectors that persist long after the assessment is published. USGCRP and author teams also identify areas where students or people early in their career may participate, for example, through youth dialogues, serving as review editors, or submitting public comments. The opportunity to meet and work with such a vast network of experts is a professional development opportunity for participants in the NCA and improves the reach of NCA’s public engagement and communication efforts (Lustig et al. 2025b). The hundreds of contributors to any given NCA form a community of communities (Avery and Crimmins 2022), providing a rich opportunity for interdisciplinary study and collaboration that benefits both authors and readers (Chu et al. 2025).

In summary, both the value and prevalence of scientific assessments in the environmental sciences may be attributed to several common factors. First, they activate scientists as “sentinels”, warning society of threats to our collective livelihoods and well-being. Second, they are situated at the science-policy interface, notably related to complex global change issues, providing a bedrock of common facts upon which policy can be informed. Third, they provide a bridge between science and action, informing decisions and responses. A final, and often underappreciated aspect of scientific assessments is the development process itself, which brings together experts across diverse areas of expertise, geographies, career stages, etc. and connects scientific communities with society.

## Guiding principles and priorities for NCA5

Effective development of climate assessments is critical to ensuring the best available science is widely accessible and widely deployed to tackle some of the world’s most challenging issues (Lubchenco 2021). For USGCRP, that means developing an assessment that “integrates, evaluates, and interprets the findings of the Program and discusses the scientific uncertainties associated with such findings; analyzes the effects of global change on the natural environment, agriculture, energy production and use, land and water resources, transportation, human health and welfare, human social systems, and biological diversity; and analyzes current trends in global change, both human-induced and natural, and projects major trends for the subsequent 25 to 100 years” (GCRA 1990). On top of that mandate, USGCRP has a goal of “continuously advancing an inclusive, diverse, and sustained process for assessing and communicating scientific knowledge on the impacts, risks, and vulnerabilities associated with a changing global climate” (USGCRP 2023b).

**Fig. 1:**
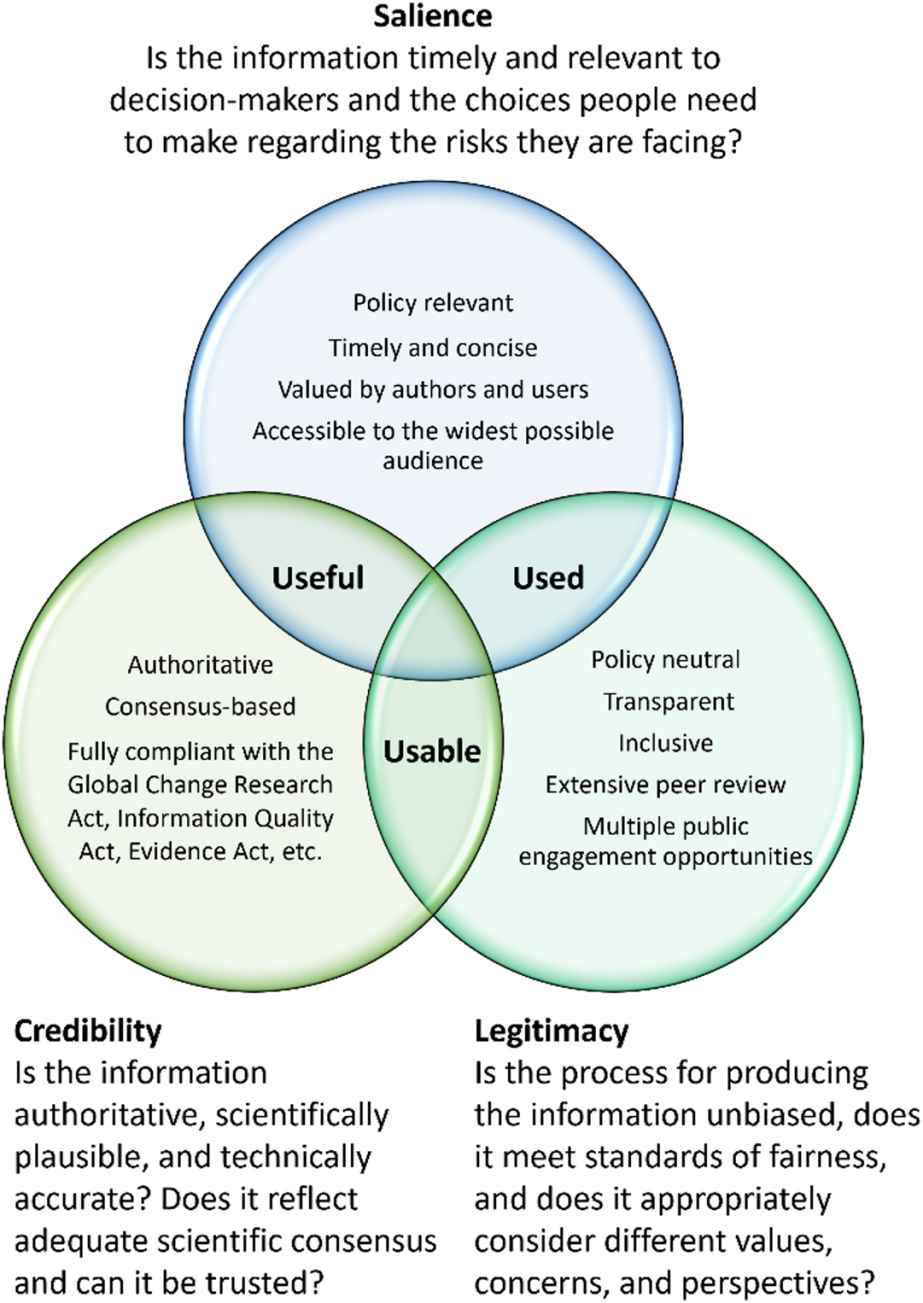
Guiding principles in the development of NCA that ensure credibility, salience, and legitimacy, or, in other words, ensure the assessments are useful, usable, and used. Adopted from Cash et al. 2002 and Lubchenco 2021

In 2020, USGCRP formalized some of the long-standing principles guiding the NCA (Avery et al. 2023c), explicitly stating that they be authoritative, timely, and concise; fully compliant with the GCRA; policy neutral, yet policy relevant; transparent and inclusive; valued by authors and users; and accessible to the widest possible audience. Each of these principles speaks to the elements of credibility, salience, and legitimacy (Cash et al. 2002), as well as the idea that assessments should be “useful, usable, and used” (Fig. 1) (Lubchenco 2021). Guided by these principles, evolution is not only necessary, it’s inevitable. Legal guidelines on the NCA change over time as new acts are introduced or revised (see supplemental materials in Avery et al. 2025); the number of people experiencing the effects of climate change increases; social norms, values, and perspectives shift; the needs of users continuously evolve, etc. As the country’s conversation on climate change advances, so too must the NCA advance to maintain its credibility, salience, and legitimacy.

Five priorities were established for the development of NCA5 (Avery and Crimmins 2022; Avery et al. 2023c). These goals overlap with each other, as well as with the guiding principles for all previous NCAs. Distilling these priorities and communicating them both internally to authors and externally to the intended audiences throughout the NCA5 cycle provided a specific framework for prioritizing advancements in the assessment development process. Many of the recent advances described in the papers within this Topical Collection can be traced back to these five priorities. Efforts to meet these priorities for NCA5 also highlight potential opportunities and challenges for future assessments.

### Priority 1: Advance the conversation

NCA5 authors were encouraged to advance the conversation on climate change by prioritizing reporting on advancements in science since the release of the fourth NCA (USGCRP 2018). With limited word count with which to draft their chapters, authors were asked to build on the findings of previous assessments but avoid repeating or re-reporting on established science. As the conversation in the United States has moved past the assertion that climate change is “real,” so, too, have the authors needed to focus on the advancing edge of science to deliver the most important information people need to make sound decisions around risks they are facing today—and will face in the decades to come.

In writing their chapters, however, NCA authors cannot *only* focus on new information. Future assessment authors cannot assume that users have read or remembered conclusions from previous assessments, and often new scientific content must be placed in context within historical findings. On the other hand, assessments will not serve their audiences if they keep growing in size with every new topic added or if readers presume that each new assessment only repeats the statements from previous reports (Brewer and McKnight 2015; The Onion 2015).

To help authors thread the needle between providing context and highlighting areas of advancement, USGCRP provided authors with background materials categorizing and summarizing the content related to their chapters that was covered in previous assessments. These preparatory materials were provided with the intent that they would help authors determine what to include by identifying where topics were previously covered or where recent advancements had been made. Between NCA4 and NCA5, USGCRP also developed a research gaps database that identified topics from previous assessments where confidence was low or more research was needed (Basile et al. 2023a). Other ways that NCA5 advanced the climate conversation was by developing downscaled data from the latest climate projections (Basile et al. 2025); encouraging diversification of information sources for the assessment by updating the information quality guidance for authors (Crimmins et al. 2024); and incorporating short focus features for the first time on topics identified by authors as timely, cross-cutting, or in areas of emerging research (Avery et al. 2023b).

### Priority 2: Make it accessible to a broad audience

This goal of NCA5—and all NCAs—involves considering how to make the assessment accessible and approachable, such that people seek out NCA as a trusted and accessible source of climate information (Jacobs et al. 2016a; Dantzker et al. 2017). This is particularly important as the assessment community moves away from developing hefty reference tomes and moves more towards assessments as a climate service: a useful and usable tool for decision-making (Buizer et al. 2013; Avery et al. 2025). Accessibility must be considered in both the development process and the assessment products. For instance, assessment developers consider how to reach broad audiences when conducting public engagement, hosting webinars and workshops, and releasing press or social media posts (NASEM 2021; Lustig et al. 2025a). In writing the assessments, authors are provided guidance and training on how to avoid jargon and use plain language. In revising the assessment, editors review the language with an eye towards maintaining readability for non-technical audiences.

A challenge in making assessments accessible is meeting the diverse needs of broad audiences, even as user needs evolve over time. Several factors make this challenge even more difficult: neither USGCRP nor any member agency has conducted a comprehensive use evaluation of NCA; evaluation is not routinely budgeted into each NCA cycle; and laws like the Paperwork Reduction Act of 1972 make it difficult to conduct user surveys or focus groups to gather user feedback (NASEM 2023; Avery et al. 2025). Some of the newly introduced processes and products by which NCA5 endeavored to meet this goal were more public engagement opportunities, closed-captioning and live-translation services during webinars and workshops, development of written and video guides on how to submit public comments in English and Spanish, and translation of the entire report into Spanish (USGCRP 2023c). Building on lessons learned from previous NCA websites, the NCA5 website developers worked to meet and exceed current digital accessibility standards (Crimmins et al. 2024), developed alternative text for every figure (Maycock et al. 2023), and optimized the site for use on phones and tablets. Additional resources like the NCA Atlas (https://atlas.globalchange.gov/), the NCA5 companion podcasts (Crimmins and Avery 2023b), and an audiobook version of the Overview chapter in English and Spanish allowed for different avenues by which users could access NCA information. A report the size of NCA has also been made more accessible to individual communities by the development of derivative materials tailored to specific audiences, both within and beyond USGCRP; examples from NCA5 include summaries (Parsons and Keenan 2023), StoryMaps (Crimmins 2023), facilitation guides (ASTC 2024), and webinar series (USGCRP 2024b).

### Priority 3: Be creative in communication

NCA5 authors were invited to leverage the power of data visualization, art, and storytelling to better communicate the climate science people can use to take action. In practice, this meant planning chapter figures early in the development process and incorporating case studies. Both figures and case studies can illuminate complex topics through examples and help readers see their neighbors taking part in climate solutions (Harold et al. 2020). Authors were aided in figure development by the National Oceanic and Atmospheric Administration Technical Support Unit (TSU), where data visualization experts turned sketches made on the backs of napkins into beautiful and information-rich images (Maycock et al. 2023). Innovations in information management and metadata collection (Waple et al. 2016; Maycock et al. 2023) have helped ensure the data behind these figures is transparent and the visualizations are reproducible.

USGCRP initiated the first-ever public call for art for NCA5, titled *Art x Climate.* This call resulted in more than 800 submissions and 92 pieces of art being included in the assessment (Lustig et al. 2025b). USGCRP also partnered with the Library of Congress to include a poem called “Startlement” at the beginning of the assessment (Avery et al. 2023a). This poem was written specifically for NCA5 by the U.S. Poet Laureate Ada Limón. In addition, the lead author of the Southeast chapter undertook an independent project with the poet laureates of Virginia and California to put out a call for poetry inspired by the publicly available draft of NCA5. They published selected poems in an award-winning anthology titled *Dear Human at the Edge of Time* (ABF 2023; Igloria et al. 2023).

### Priority 4: Make it about people

Closely tied to making the assessment accessible to wide audiences, NCA5 authors were encouraged to think about how to make the American people feel seen by their chapters and how to write a report that best serves user needs. Part of making NCA broadly applicable and representative of people across the United States is making sure NCA is written by a diversity of authors from around the country (Jacobs et al. 2016a). This involved establishing an expectation that diversity was valued by USGCRP, updating guidance for lead authors on how to select diverse author teams to reflect modern understanding of the many personal and professional axes encompassed in the term “diverse”, and instituting a code of conduct so that participants would feel safe voicing their perspectives (NASEM 2021; USGCRP 2023b; Chu et al. 2025). Diverse author teams also resulted in a broader reach when it came to public engagement, as authors extended communication about engagement opportunities across their networks (Lustig et al. 2025a).

Public engagement efforts help ensure that NCAs represent the American public and their needs by providing multiple means for people to weigh in on the process and products (Cloyd et al. 2016) and to observe the ways in which authors respond to feedback (for example, see responses to public comments at https://nca2023.globalchange.gov/downloads). One outcome of feedback received from the public and USGCRP’s Social Science Task Force, beginning in at least 2012 and extending into the NCA5 process, was to incorporate more social science information, tools, and frameworks (Weaver et al. 2017; Roesch-McNally et al. 2020; Maxwell et al. 2022). This resulted in two new chapters in NCA5, one on Economics (Hsiang et al. 2023) and one on Social Systems and Justice (Marino et al. 2023). NCA5 also had a high number of social scientists participating and included information on climate impacts on culture, heritage, and traditions (Maxwell et al. 2025). To aid cross-report integration of equity and environmental justice topics, NCA5 authors were provided with training on these topics, as well as on the use of Indigenous Knowledge. In addition to these supportive practices and resources, NCA5 was better positioned than previous NCAs to include equity and justice topics, as the research in these fields had advanced significantly since 2018 and shifts in public consciousness have aided general understanding of how climate change and social justice are interconnected (Méndez et al. 2025).

### Priority 5: Ensure it is useful and usable

Many of the advancements described above speak to this last priority of NCA5 in some way, whether it is by making the topics of the report more relatable, writing in accessible language, developing new means of accessing the information (e.g., podcasts), or improving website user experience. NCA5 also continued the use of risk-based framing adopted by previous assessments to ensure the chapters first address the things that people value and then demonstrate how climate change is putting those things at risk (NASEM 2021; Avery et al. 2025). As the NCA has grown in size, it has become increasingly important for chapter leadership to take on the responsibility of making connections across chapters (Conrad-Rooney et al. 2025) and to create links that allow readers to quickly find the information they need across the report.

NCA5 authors were also reminded that while climate experts like themselves might be one subset of the larger NCA audience, the primary targeted audience is a much broader set of decision-makers facing critical risk-management choices. USGCRP seeks to avoid producing an NCA written by scientists for other scientists (Crimmins and Avery 2023a). Instead, USGCRP —for several iterations of NCA now— has placed a premium on integrating processes to democratize climate data in their products—by making datasets and processes accessible through metadata and by providing findings in accessible language and a range of formats (e.g., text, figures, factsheets, presentations, podcasts, etc.). The goal has been to enable users to reproduce the findings of the report independently, adding to the transparency and credibility of the final product. NCA5 initiated the development of the NCA Atlas (https://atlas.globalchange.gov/), which allows users to develop their own climate maps based on NCA data. Highlighting case studies and voices of community members (Huntington et al. 2023) and incorporating artists’ perspectives (Lustig et al. 2025b) were intended to help readers place themselves within their own climate stories and encourage those outside the scientific community to feel like they belong in the country’s climate conversations. Expanding the idea of who assessments are developed by, about, and for can also improve readers’ understanding that climate change is not just something happening to others, far away and in the future. Climate risks, impacts, and responses are indeed things everyone living in the United States is facing today (Jay et al. 2023).

## Future of climate assessments

Climate change is increasingly a priority of focus for decision-making in public, private, and non-governmental sectors, as well as at the municipal, business, or household levels. Climate assessments have become a significant and impactful means of providing useful and usable scientific information to decision-makers in actionable formats (Oppenheimer et al. 2019). It seems likely that assessment efforts to synthesize, distill, and describe climate risks, impacts, and responses will only become more necessary to support societal choices as the world continues to face multiple global challenges. In revisiting assessment history and observing the current trajectory of climate assessments, we note that some trends in the evolution of the assessment development process are already visible. Below we identify three such trends, which highlight near-term opportunities and challenges in assessment development.

### Growing demand for context-specific information

Over the past 30+ years of climate assessments, global change science has continued to advance and reveal greater insight into how humanity is altering the Earth system. When the first IPCC Assessment Report was developed in 1990, the scientific community was focused on global-scale Earth system modeling and climate projections. As observational records have expanded and computational power has increased, understanding of Earth system processes has vastly improved. With these advances in climate science, the questions being asked by science users-- and the information researchers need to answer those questions-- have shifted user demand toward more local or more context-specific climate data. These include, for example, small geographic areas with complex biophysical conditions or microclimates, or communities with unique socioeconomic vulnerabilities (Basile et al. 2024). Furthermore, advances in sociology have improved understanding of human behavior around mitigation, adaptation, and resilience (Marino et al. 2023). Since climate assessments synthesize existing literature, expertise, and a range of other sources of information, they have naturally followed a similar pathway of evolution. Understanding social impacts and responses are important elements of connecting knowledge to action, and thus demand for increased coverage of social science in climate assessments is expected to continue.

A sustained two-way exchange of information between national assessment developers and state and local assessment developers would improve climate services, empower decision-making across geographic scales, and accelerate the realization of a truly climate-ready nation. Following a similar pattern observed in international and national assessments, municipalities are turning towards state and local climate assessments for hyper-localized and site-specific information to support decision-making (California 2018; Adams et al. 2021; Wisconsin 2021; Wuebbles et al. 2021; Bonventre and Lester 2022). The resources the U.S. federal government has invested in developing a robust NCA enterprise and growing an assessment community can help inform and build assessment and technical support expertise at the local level. Development of future NCAs is likely to occur in parallel to a rising need for resources, capacity-building, and guidance in support of local assessment efforts. Future NCAs could be an epicenter for a broader network of climate assessments at varying geographic scales and timely, topical special reports, and could serve as a foundation for a multitude of climate services (Moss et al. 2019).

At the same time, state, local, and Tribal assessments have adopted innovative practices that reflect a more curated response to user needs than many national assessments have been able to attain within their mandated time frames. These include processes like knowledge co-production, better incorporation of Indigenous Knowledge (STACCWG 2021; 2025), and funding research on gaps in scientific information identified through the assessment feedback process (Matouka and Basile 2025). Lessons learned from these subnational assessment practices can serve as valuable information for national and international assessment developers.

### Incorporating diverse scientific knowledge into climate assessments

A major advance of recent climate assessments is a more thoughtful and comprehensive integration of information from diverse knowledge systems (Ford et al. 2016). Diverse perspectives and epistemologies, including Indigenous Knowledges, have been included in previous NCAs and have advanced with each new report. Notably, the information quality guidance to authors was revised in 2012, providing further consideration for Indigenous Knowledges ahead of NCA3 (Waple et al. 2016), and authors of NCA4’s Tribes and Indigenous Peoples chapter developed advice for their fellow authors on writing about Indigenous peoples. NCA5 updated the 2012 information quality guidance for authors alongside the development of federal guidance (OSTP 2022), codified the NCA4 authors’ advice on writing about Indigenous peoples into the official NCA5 author guidance, provided training to authors and staff on how to respectfully and responsibly use and integrate these information sources into their chapters, and hosted an informational webinar to agency staff tasked with reviewing the NCA to ensure they were prepared to review content that incorporated Indigenous Knowledges (STACCWG 2021; Crimmins et al. 2024; Whyte et al. 2025).

In addition to Indigenous Knowledges, NCA5 included perspectives from local expert knowledge and lived experiences (Huntington et al. 2023), environmental justice scholarship (Méndez et al. 2025), and expanded coverage of concepts and frameworks from the social sciences and humanities (Chu et al. 2025; Maxwell et al. 2025). The inclusion of art and poetry in NCA5 also showcased new and valuable ways of understanding, interpreting, and imagining climate change (Lustig et al. 2025b).

Incorporation of diverse knowledge systems and perspectives in climate assessments, as well as community-driven science, will continue to be needed to fully describe and understand the impacts and responses of climate change (Galford et al. 2016). The broadening of epistemologies and knowledge bases in climate assessments has led to important, yet often controversial questions such as: Who is considered a climate expert? How is it decided (and who decides) what is authoritative and trustworthy information that can or should be included in a climate assessment? How should guidance for compliance with laws such as the Information Quality Act (2000) and the Evidence Act (H.R.4174 2018) be updated to respectfully include broader knowledge bases in climate assessments while still adhering to the Federal Advisory Committee Act (FACA 1972)? As climate assessments expand to include new and timely information and ideas, how should the expertise of the editorial, technical, and peer reviewers expand to adequately evaluate content not covered in previous assessments?

Because scientific credibility is a cornerstone of the climate assessment process, it is critical for developers of climate assessments who aim to broaden knowledge bases and perspectives to continue to be open and transparent with how data and findings are selected, vetted, and described. It is incumbent upon assessment developers to make clear to the reader, through processes such as metadata documentation and traceable accounts, where a given statement or finding originates (Avery et al. 2025; Champion et al. 2023) and to provide options for multiple levels of comprehension or interaction (e.g., skimming key takeaways, reading chapter content for deeper understanding of a specific topic, applying the methods in metadata records to recreate figures, or using datasets and computer code in external research or analyses).

As developers look towards scientific disciplines that have not been well represented in previous assessments, maintaining the extremely high level of scientific credibility established in assessments like NCA and IPCC will entail working closely with the knowledge holders within those fields to understand how expertise, authority, and information quality is established in a given epistemology or knowledge system (OSTP 2022). There are some existing tools that can guide this understanding across disciplines, including principles like FAIR (findability, accessibility, interoperability, and reusability), systems that allow for traceability like the Global Change Information System (https://data.globalchange.gov), and other Federal guidance and best practices (Crimmins et al. 2024). However, as described by (Maxwell et al. 2025), achieving interdisciplinary integration in climate assessments entails altering knowledge governance, likely beyond the existing guidance and principles governing NCA (Maldonado et al. 2016; Obermeister 2017).

### Accessible and creative communication

In the early years of climate assessments, a primary objective was putting forward authoritative sources of information about the state of the science around climate change. While that remains a key goal, the assessment audience has grown well beyond the climate science community and a few select policymakers. Meanwhile, the demand for a “state of the science” report has evolved into more sophisticated calls for decision-support tools and other climate services (NASEM 2021; NSTC 2023). In response to evolving demands, climate assessment developers have evolved the suite of offerings they provide beyond just a written report. For example, NCA4 published a website where the report’s downscaled climate projections were made available. Building on this, NCA5 developed a complementary atlas that enables users to find location-specific, high-resolution data through interactive exploration of observed and projected climate variables over a range of scenarios and future warming levels. Such interactivity is intended to empower assessment users to easily create bespoke maps and datasets with highly customizable climate information (USGCRP 2023a).

NCA5 products took multiple media forms: websites, figures, printable chapters and handouts, interactive maps, podcasts, audiobooks, visual art, poetry, engagement workshops, webinars, social media, etc. With the exception of short tutorial videos (e.g., how to submit public comments, how to use the NCA Atlas) and recorded webinars however, video-storytelling remains an underutilized tool for NCA. High-quality videos are powerful mediums for sharing knowledge and information. As seen with Art x Climate (Lustig et al. 2025b), visual content can engage viewers in new climate conversations, build a more nuanced understanding of how others are experiencing climate impacts, and forge connections for people to understand their own climate risks. Development of audiovisual content can also be a powerful learning tool for the producers of videos themselves. Just as authors with different expertise and perspectives realize a shared vision and narrative while writing chapter drafts together, the storytelling process involved in co-production of audiovisual content can result in authors’ deeper understanding of their own disciplines.

Numerous barriers make video production difficult for NCA developers. For instance, limited resources are available to building out video production capacity, whether for the NCA content itself or for post-production communication videos. While the TSU supports graphic and technical design of NCA figures and websites, creating videos has been outside the scope of the TSU’s purview and funding agreements. For a video to be included or embedded into an NCA chapter, additional guidance would need to be established early in an assessment development cycle, ideally prior to onboarding authors. Decisions would need to be made by USGCRP on questions of video provenance, production, copyright permissions, and dissemination. The NCA steering committee would also need to create and update author guidelines on this new form of chapter content development, including measures to ensure information quality and accessibility. A process would need to be established for incorporating video transcripts and storyboards into formal internal reviews, peer reviews, and public comment periods. Finally, tasks associated with video production, editing, and formatting would need to be added to the TSU’s product requirements. Many of these barriers meant that videos produced by The Story Group after publication of NCA3 were not able to be considered official NCA content (The Story Group 2014).

Despite these challenges, one NCA5 author team took steps towards incorporating video content in their chapter, which may inform the creation of new assessment development processes. Using their own volunteered human and technological resources, authors from the NCA5 Human Health chapter developed video interviews with Tribal leaders in Louisiana. These leaders spoke about the health impacts they experienced from extreme weather events and the climate adaptation actions taken by their community to uphold sovereignty and self-determination (CRT 2023; Hayden et al. 2023). Documenting oral accounts of lived experiences is one example of incorporating diverse knowledge into assessments. The direct narratives and wisdom shared in these videos met the information quality requirements as sources of Indigenous Knowledges and local lived experience and, thus, could be included as source materials to inform NCA findings (Champion et al. 2023). However, without the established guidance and processes noted above in place to embed videos into the report, the Human Health chapter could only provide external links to these resources (CRT 2023) and the video content was not included in the official assessment delivered to Congress (Avery et al. 2025). This likely resulted in fewer views or less use of the videos than if they’d been embedded in the NCA5 chapter’s website or used in NCA5 communication and outreach materials. Future assessment developers will need to revisit these procedural issues, as videos and other multimedia products are valuable tools for science communication and storytelling.

## Conclusion

Assessment developers at the local, state, Tribal, regional, national, and international levels are faced with the continuous challenge of meeting evolving user needs. Such responsiveness is imperative to the continued success of assessments themselves as credible, salient, and legitimate sources of useful and usable scientific information. It is also imperative to ensure assessments continue to inform societal choices on how to address global crises, manage risks, and build resilience.

In addition to the insights and recommendations found within the suite of papers in this Topical Collection, several considerations for future climate assessment developers can help address the increasing need for decision-relevant information on climate impacts, risks, and responses. These include continued and strengthened conversations between international, national, state, local, and Tribal assessment developers to share innovations and lessons learned in the development process; working with knowledge holders in under-represented areas of expertise to alter assessment governance and guidelines and better incorporate diverse perspectives; and seizing opportunities to provide improved climate services through innovative communication and engagement mediums.

Many of the recommendations outlined in this paper, and in the other papers within this Topical Collection, also support the USGCRP’s 2022−2031 Strategic Plan’s goals and priorities (USGCRP 2022), which are aimed at accelerating systems-based research and delivering the useful, accessible, and inclusive data needed to inform decision-making in a rapidly changing world.

## Data Availability

No original datasets were generated or used in this paper. All referenced datasets are linked and available in reference lists.
